# Genomic-Wide Analysis of the PLC Family and Detection of GmPI-PLC7 Responses to Drought and Salt Stresses in Soybean

**DOI:** 10.3389/fpls.2021.631470

**Published:** 2021-03-03

**Authors:** Zhi-Feng Chen, Jing-Na Ru, Guo-Zhong Sun, Yan Du, Jun Chen, Yong-Bin Zhou, Ming Chen, You-Zhi Ma, Zhao-Shi Xu, Xiao-Hong Zhang

**Affiliations:** ^1^College of Life Sciences, Northwest A&F University/State Key Laboratory of Crop Stress Biology for Arid Areas, Yangling, China; ^2^Institute of Crop Science, Chinese Academy of Agricultural Sciences (CAAS)/National Key Facility for Crop Gene Resources and Genetic Improvement, Key Laboratory of Biology and Genetic Improvement of Triticeae Crops, Ministry of Agriculture, Beijing, China

**Keywords:** PLC proteins, genomic-wide analysis, abiotic stresses, hairy root assay, *Glycine max*

## Abstract

Phospholipase C (PLC) performs significant functions in a variety of biological processes, including plant growth and development. The PLC family of enzymes principally catalyze the hydrolysis of phospholipids in organisms. This exhaustive exploration of soybean GmPLC members using genome databases resulted in the identification of 15 phosphatidylinositol-specific PLC (GmPI-PLC) and 9 phosphatidylcholine-hydrolyzing PLC (GmNPC) genes. Chromosomal location analysis indicated that GmPLC genes mapped to 10 of the 20 soybean chromosomes. Phylogenetic relationship analysis revealed that GmPLC genes distributed into two groups in soybean, the PI-PLC and NPC groups. The expression patterns and tissue expression analysis showed that GmPLCs were differentially expressed in response to abiotic stresses. *GmPI-PLC7* was selected to further explore the role of PLC in soybean response to drought and salt stresses by a series of experiments. Compared with the transgenic empty vector (EV) control lines, over-expression of *GmPI-PLC7* (OE) conferred higher drought and salt tolerance in soybean, while the *GmPI-PLC7*-RNAi (RNAi) lines exhibited the opposite phenotypes. Plant tissue staining and physiological parameters observed from drought- and salt-stressed plants showed that stress increased the contents of chlorophyll, oxygen free radical (O_2_^–^), hydrogen peroxide (H_2_O_2_) and NADH oxidase (NOX) to amounts higher than those observed in non-stressed plants. This study provides new insights in the functional analysis of *GmPLC* genes in response to abiotic stresses.

## Introduction

Plants inhabit widely varying environments and are affected by abiotic and biotic factors such as water, temperature, herbivore attacks and disease threats. Drought and salt are important factors that affect the production of crops because these two environmental stresses significantly impact plant reproduction. Drought- and salt-stressed soybean, for example, produce fewer pods and seeds per plant, and consequently reduce its yield and economically diminish the industrial production of soybeans (Wang et al., 2015a). In recent years, crop scientists have become very interested in the study of signal transduction pathways related to plant stress response. A particular focus has been on phospholipase C (PLC) because it is a very important enzyme in signal transduction pathways; many experts and scholars have directed their research on PLCs in association with plant tolerance to stress.

In response to various stresses, diverse signaling pathways are activated in plants, including Calcium ion (Ca^2+^), protein phosphatase, protein kinase and lipid signaling cascades. At present, plant lipid signal transmission is a relatively important area of research, including phosphatidic acid (PA) signaling (Berridge, 1993; Meijer and Munnik, 2003). Different PA generators are activated in stress response and PLCs contribute to PA response to stress (Meijer and Munnik, 2003). Phospholipases can be roughly divided into four categories, phospholipase A1 (PLA1), phospholipase A2 (PLA2), PLC and phospholipase D (PLD) (Hong et al., 2016), based on the different cleavage sites of phospholipids that are hydrolyzed by a phospholipase. As a key enzyme in signal transduction pathways, PLC mainly catalyzes the hydrolysis of phospholipids in organisms.

The PLC-mediated signaling pathway is one of the classic pathways of cell signaling pathways (Kadamur and Ross, 2013). In plants and animals, the PLC family is a multi-gene family whose genes have various functions (Nakamura et al., 2005; Peters et al., 2010). Based on their affinities to different substrates, PLCs are divided into two major groups in plants, phosphatidylinositol-specific PLC (PI-PLC) (Vossen et al., 2010) and phosphatidylcholine-PLC (PC-PLC), the latter of which basically hydrolyze phosphatidylcholine (PC) and other lipids and is also called non-specific phospholipase C (NPC) (Nakamura et al., 2005; Peters et al., 2010). Although the structures of certain PI-PLCs have been described in plants, knowledge about the structure of NPCs is lacking.

Phosphatidylinositol-specific PLC specifically recognizes phospholipids and consists of a catalytic PI-PLC-X (PF00388) domain, PI-PLC-Y (PF00387) domain, Ca^2+^/phospholipid-binding C2 domain (PF00168) and EF hand-like domain (PF09279). These structures of PI-PLC are present in all organisms and exhibit minimal differences in performing different functions (Rupwate and Rajasekharan, 2012). The EF chiral domain is located at the N-terminus of the PI-PLC sequence and contains four helix-loop-helixes which specifically recognize and bind to its substrate phosphatidylinositol 4,5-bisphosphate (PIP2) (Nomikos et al., 2015). When plants are subjected to external stresses, the EF chiral domain regulates the function of the reduced coenzyme II oxidase respiratory burst oxidase homolog protein D (RBOHD), an oxidase of nicotinamide adenine dinucleotide phosphate (Yasuhiro et al., 2015). The C2 domain is located at the C-terminus of PI-PLC and mainly functions to combine with Ca^2+^ to trigger the hydrophobicity of PLC, and thus it improves the hydrolysis efficiency of phospholipids (Hunt et al., 2004). The X and Y catalytic domains are located between the N-terminus and C-terminus of the PLC sequence. They are the two most conserved domains of PLC and are the crucial domains of PLC’s catalytic function. These domains can also mediate PLC-targeted membrane localization by electrostatic interactions (Nomikos, 2015).

The first functional *PI-PLC* cloned from plants was from *Arabidopsis* (Hirayama et al., 1995). According to genomic analyses, there are nine PI-PLCs and six NPCs in *Arabidopsis* (Pical, 2002; Jessica et al., 2004; Tasma et al., 2008). Additionally, six *PI-PLC* members have been identified in tobacco (Nakamura et al., 2005), and many more PLCs have been found in other plants (Mcmurray and Irvine, 1988; Einspahr et al., 1989; Hirayama et al., 1995; Shi et al., 1995; Song and Goodman, 2002; Venkataraman et al., 2003; Yun et al., 2004; Das et al., 2005; Zhai et al., 2005; Dowd et al., 2006).

Phospholipase C is involved in biotic and abiotic stress response in plants (Luo et al., 2005; Kalachova et al., 2016). For example, the expression of *AtPLC1* in *Arabidopsis* can be induced by abiotic stresses, such as low temperature, drought and salt (Hirayama et al., 1995). There is a differential requirement of PLC for the tomato immune response and that SiPLC4 is specifically required for Cf-4 function, while SiPLC6 may be a more general constituent of the hypersensitive response (HR) protein signaling (Vossen et al., 2010). PLC pathway participates stress-induced Ca^2+^ signals and confers salt tolerance to rice (Li et al., 2017). In addition, the basal level of expression of most *Arabidopsis* dehydration resistance element binding protein 2 (*DREB2*) genes were negatively regulated by *PI-PLC*. The *DREB2* genes play important roles in plant response to environmental stresses, including dehydration (Liu et al., 1998; Ruelland et al., 2013). One of the most important mechanisms by which a plant responds to signals of abiotic stress is to induce the expression of stress-related genes (Kalachova et al., 2016). Under drought stress, the transcription levels of *StPLC1* and *StPLC2* in potato leaves are elevated, while the level of *StPLC3* are unchanged (Kopka, 1998). Under drought stress, the expression levels of *TaPLC1* in wheat rapidly increased compared to that of *TaPLC1* under the non-drought treatment (Zhang et al., 2014). These different expression levels indicated that different *cis*-elements and trans-acting factors might regulate the expression levels of phospholipase C homologs and that various PLCs have specific functions. In recent years, some studies have shown that PLC is also involved in the regulation of hormone signaling pathways in various plants (Meijer and Munnik, 2003; Kalachova et al., 2016). In *Arabidopsis*, PLC promoted ABA-induced stomatal closure; the PLC specific inhibitor U73122 can inhibit ABA-induced stomatal closure and changes in Ca^2+^ content (Drøbak, 1992). In addition, NPC, a non-specific plant phospholipid enzyme, is homologous to bacterial PI-PLC and can response to lipid signals (Pokotylo et al., 2013). Especially in recent years, NPC family research has made significant progress, which involves various aspects of plant growth and development (Nakamura and Ngo, 2020).

Soybean (*Glycine max*) is an important oil and protein crop whose growth and productivity are adversely affected by soil drought and salt (Wang et al., 2015b). In addition, soybean seed is the most important protein source used to feed farm animals. It represents two-thirds of the total world output of protein feedstuffs (Whitham et al., 2016). Previous studies highlighted the role of *PLC* genes in plant growth and development and that several *PLC* genes were involved in abiotic stresses. Although the *PLC* gene family has been analyzed in several plant species, no systematic investigation has been conducted using soybean, and little information is available about the function of *GmPLCs* in abiotic stress responses. Given the significance of this gene family, a genomic-wide identification of *GmPLCs* was performed in the current study, including investigations into phylogenetic relationships, chromosomal locations, gene duplications and expression profiles of these genes. In addition, we determined the function of *GmPI-PLC7* in soybean. The results indicated that over-expression (OE) of *GmPI-PLC7* increased soybean tolerance to drought stress. Our study provides insights into understanding the genetic basis of *GmPLC* in abiotic stress response.

## Materials and Methods

### Identification of Soybean GmPLC Members

Identification of the soybean *PLC* gene family was performed according to the method described previously (Amarjeet et al., 2013; Su et al., 2020), with some revisions. The putative *GmPLC* gene family members were identified in the soybean gene family databases Phytozome v12 and SoyBase (Goodstein et al., 2012), using keywords such as “phospholipase C,” “phosphatidylinositol phospholipase C,” and “phosphoesterase.” We verified identifications using the phospholipase C domain in the SMART database (Letunic et al., 2012). Hidden Markov Model profiles using default parameters (*E*-value < e−20) were acquired for different PLC classes from the Simple Modular Architecture Research Tool (SMART) database and used to scan the protein database Soy Base (Finn et al., 2011). *Arabidopsis* PLCs were obtained from TAIR (The *Arabidopsis* Information Resource) and were used to BLASTP soybean homologous sequences in Phytozome v12. The putative GmPLC sequences having higher homology with *Arabidopsis* PLC were obtained for further analysis. After the integration of results from all these databases, unique sequences (with unique locus IDs) were selected to remove redundancies. All the sequences were verified to have the conserved domains and motifs from relevant sequences in the Inter Pro and Pfam databases (Axelrod et al., 2010).

### Chromosomal Location and Phylogenetic Analysis

The position information of *GmPLCs* on the soybean chromosome were obtained from the Phytozome database and were visualized with the online tool Map Gen 2 Chromosome v2. For the phylogenetic analysis, the protein sequences of the verified PLC candidates from rice, *Arabidopsis* and soybean were used for multiple sequence alignment by employing Clustal X (version 2.0). An un-rooted phylogenetic tree was constructed to analyze the evolutionary relationships of the *PLC* genes. The phylogenic tree was constructed by MEGA 7.0 software with the neighbor-joining (NJ) method. We estimated the confidence levels with bootstrap analyses of 1000 replicates, and the default values were set for all the parameters. We didn’t use outlier to construct the tree and didn’t find the setting of outlier in MEGA7.0 software.

### Multiple Sequence Alignment and Conserved Motifs Analysis

Position and sequence information of *GmPLC* genes were used to align sequences using the DNAman software and Multiple Em for Motif Elicitation (MEME). The coding sequences and the genomic DNA sequences of GmPLCs were obtained from SoyBase. The exon/intron gene boundaries were examined using the Gene Structure Display Server 2.0 (GSDS) tool (Hu et al., 2015).

### Tissue-Specific Expression Patterns of *GmPLC* Genes in Soybean

Transcription data were obtained from Phytozome v12 to analyze the tissue expression patterns of *GmPLCs*. HemI software was used to visualize the hierarchical clustering of all the *GmPLCs*.

### Promoter Sequence Analysis

The 2.0-kb 5′ sequences upstream of GmPLC genes were extracted from the Phytozome database as regulatory promoter regions. Putative *cis*-acting elements were analyzed using the Plant CARE database (Rombauts et al., 1999). The *cis*-acting element boundaries were examined using the GSDS tool (Hu et al., 2015).

### Plant Materials and Treatments

Soybean (Williams 82) was used to analyze the expression pattern of *GmPLC* genes. Seedlings were grown in pots in a greenhouse with a 16 h-light/8 h-dark photoperiod, 28/20°C day/night temperatures, and 70% relative humidity. The 14-day-old seedlings were subjected to drought, mannitol (to simulate osmotic stress) and melatonin treatments. For the drought treatment, soybean plants were removed from soil and put on filter paper; for the mannitol treatment, the roots of soybean were soaked in 150 μM mannitol solution; and for the melatonin treatment, the leaves were subjected to 150 μM melatonin solution. The leaves of seedlings were collected at 0, 0.5, 1, 2, 4, 8, 12, 24, and 48 h after initiation of treatments. All samples were frozen expeditiously in liquid nitrogen and then stored at −80°C for RNA extraction (Xu et al., 2008).

### RNA Extraction and Quantitative Real-Time PCR

Total RNA was extracted from soybean leaves of seedlings subjected to one of the three treatments described above using the manufacturer’s protocol (TIANGEN, China), and the RNA was treated with DNase I (TaKaRa, Japan) to exclude genomic DNA contamination, according to the manufacturer’s instructions. Approximately 3 μg of purified total RNA from each sample was used in reverse transcription using TransScript One-Step gDNA Removal and cDNA Synthesis Super Mix (TransGen Biotech, Beijing, China) and then samples were kept at −20°C. The Quantitative Real-Time PCR (qRT-PCR) was performed with Super Mix (TransGen Biotech, Beijing, China) on an ABI Prism 7500 system (Applied Biosystems, Foster City, CA, United States), and each qRT-PCR reaction was repeated three times. Data analysis was conducted by the 2^–ΔΔCT^ method. The primers used for qRT-PCR are listed in Supplementary Tables 1 **and** 5.

### *Agrobacterium rhizogenes*-Mediated Transformation of Soybean Hairy Roots

We generated *GmPI-PLC7*-over-expressing (*GmPI-PLC7*-OE) soybean hairy roots. The sequences of *GmPI-PLC7* were assembled with the plant transformation vector *pCAMBIA3301* and the *CaMV 35S* promoter. For the construction of the RNAi vector, a 463-bp fragment including the first intron sequence and its reverse complement sequence were synthesized (Biomed, Beijing, China) and cloned into *pCAMBIA3301* to generate the *pCAMBIA3301-GmPI-PLC7*-RNAi vector. All recombinant vectors were transformed into soybean hairy roots by the *A. rhizogenes*-mediated method as described previously (Wang et al., 2009; Du et al., 2018). The injected plants were transferred to a greenhouse with high humidity until the hairy roots were produced at the infection site. The original main roots were cut off from 0.5 cm below the infection site. Seedlings were transplanted into fertilized soil and cultured in the greenhouse for a week at 25°C and a 16 h light/8 h dark photoperiod (Kereszt et al., 2007). After verification, positive soybean hair roots were used for abiotic stress assays, and we have changed references with more detailed methods descriptions of transformed Soybean Hairy Roots. There were five lines in a pot and six biological replicates of each stress treatment.

### Drought and NaCl Tolerant Assays

For the drought treatment, 2-week-old soybean plants with transgenic hairy roots were subjected to dehydration for 16 days. For the salt treatment, 2-week-old soybean plants with transgenic hairy roots were treated with 250 mM NaCl solution for 7 days (Wang et al., 2017).

### Measurement of Proline, Malondialdehyde (MDA), Oxygen Free Radical (O_2_^–^) and Hydrogen Peroxide (H_2_O_2_) Contents

The contents of proline, MDA, and H_2_O_2_ and O_2_^–^ were assessed in transgenic, GmPI-PLC7-OE (OE), empty vector (EV), and GmPI-PLC7-RNAi (RNAi) lines of plants with the corresponding assay kit (Cominbio, Suzhou, China) based on the manufacturer’s protocols (Du et al., 2018).

### Leaf-Staining by 3,3-Diaminobenzidine (DAB), Nitro Blue Tetrazolium (NBT) or Trypan Blue

After drought treatment for 7 days or salt treatment for 3 days, leaves from transgenic soybean plants were used for staining. For the DAB staining, the samples were immersed in DAB solution (Solarbio, China) for 18 h and then transferred to a boiled solution of a ratio of alcohol to glycerin of 3:1 until leaves became white in color. For the NBT staining, the samples were immersed in NBT staining solution (Solarbio, China) for 14 h and then transferred to a boiled solution of three-parts alcohol to one-part glycerin to decolor the leaves to a white color (Du et al., 2018). For the trypan blue staining, plants were subjected to drought for 7 days, and then the samples were immersed in 0.4% trypan blue (Solarbio, China) solution for 12 h followed by decoloring in acetic acid until the leaves became white.

### Statistical Analysis

All treatments in each of the phenotypic measurements and qPCR expression analysis described above had at least three independent replicates. Values are means ± standard deviations (SD) and variance analysis was performed based on the Student’s test at *p* = 0.05.

## Results

### Identification of GmPLCs in Soybean

Twenty-four GmPLCs, were identified based on the unique conserved domains (*X* and *Y* domains). The sizes and physical and chemical properties varied among different GmPLC proteins. The statistical results illustrated that the protein size of 15 GmPI-PLC proteins ranged from 376 to 629 amino acids, the isoelectric points (p*I*) varied from 5.70 to 9.45, and the molecular weights (MW) ranged from 42.7 to 70.9 kDa, whereas the protein lengths of nine NPCs ranged from 311 to 801 amino acids, the p*I* varied from 5.23 to 7.78 and the MW ranged from 33.4 to 59.7 kDa. The 15 GmPI-PLCs and 9 GmNPCs were named as *GmPI-PLC1* to *GmPI-PLC15* and *GmNPC1* to *GmNPC9*, respectively, according to their chromosomal locations (Supplementary Table 2).

### Chromosomal Location and Phylogenetic Analysis

**FIGURE 1:**
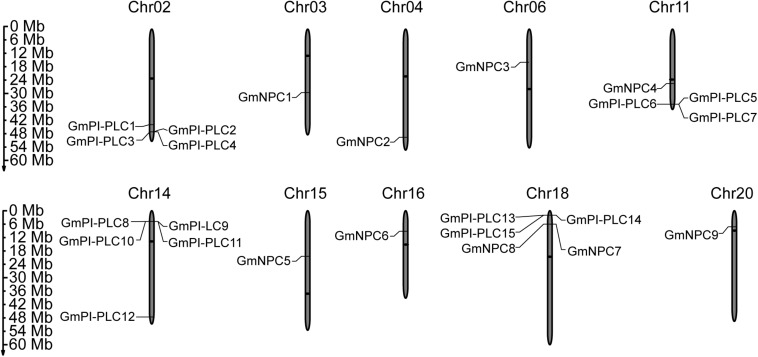
Chromosomal distribution of the identified 15 *GmPI-PLC* genes and 9 *GmNPC* genes in soybean. The members of *GmPLC* genes were distributed on different chromosomes (Chr). The gray bars represent the Chrs, and the Chr numbers are shown above the bars. Bars are not drawn to scale. The numbers on the left side of the bars show the distances in megabases (Mb) between neighboring genes.

The PLC genes were distributed on 10 of the 20 total chromosomes in soybean. Soybean chromosome 14 contained the most *GmPI-PLCs*, whereas chromosomes 2, 11 and 18 contained the comparable number of *GmPI-PLC* genes. *GmNPC* genes were evenly distributed across nine of the chromosomes (Figure 1).

**FIGURE 2:**
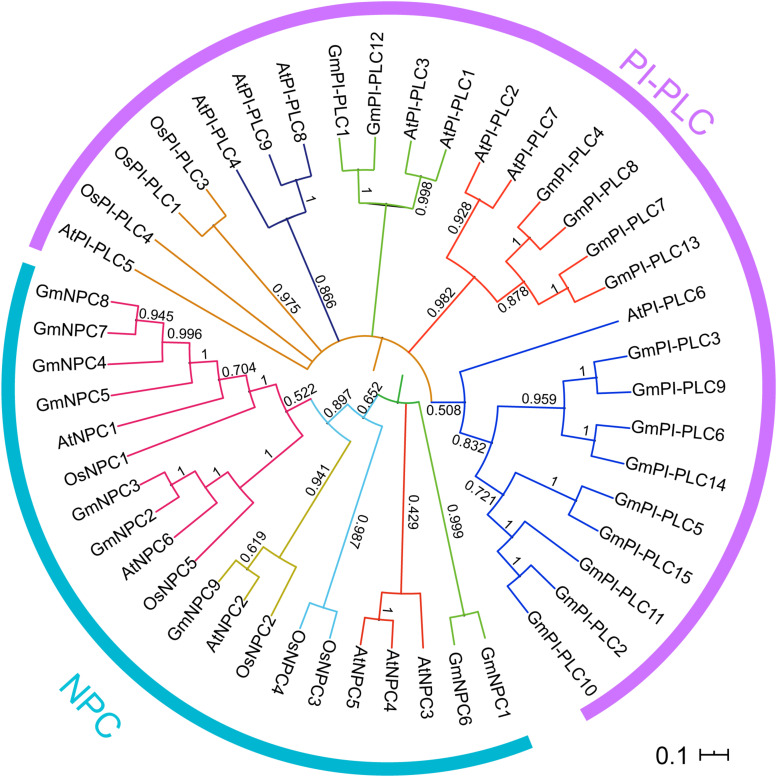
Phylogenetic relationships of the *PI-PLC* and *NPC* gene families in soybean, rice and *Arabidopsis*. The complete amino acid sequences of the PLC proteins were aligned by Clustal W and maximum-likelihood estimation with MEGA7. Two discrete groups are highlighted in different colors.

To analyze the evolutionary relationships of PLC proteins, the PLC proteins from soybean and other organisms were aligned and used for a phylogenetic analysis. All of 24, 14, and 9 PLCs from soybean, *Arabidopsis* and rice, respectively, were used for the construction of the phylogenetic tree through the MEGA 7.0 software (Figure 2). The PLC proteins in soybean could be grouped as two distinct clusters GmPI-PLC and GmNPC, and 15 GmPI-PLC proteins were divided into three major clades, which corresponds to phylogenetic results in previous reports (Supplementary Figure 1 **and** Table 3).

### Multiple Sequence Alignment

**FIGURE 3:**
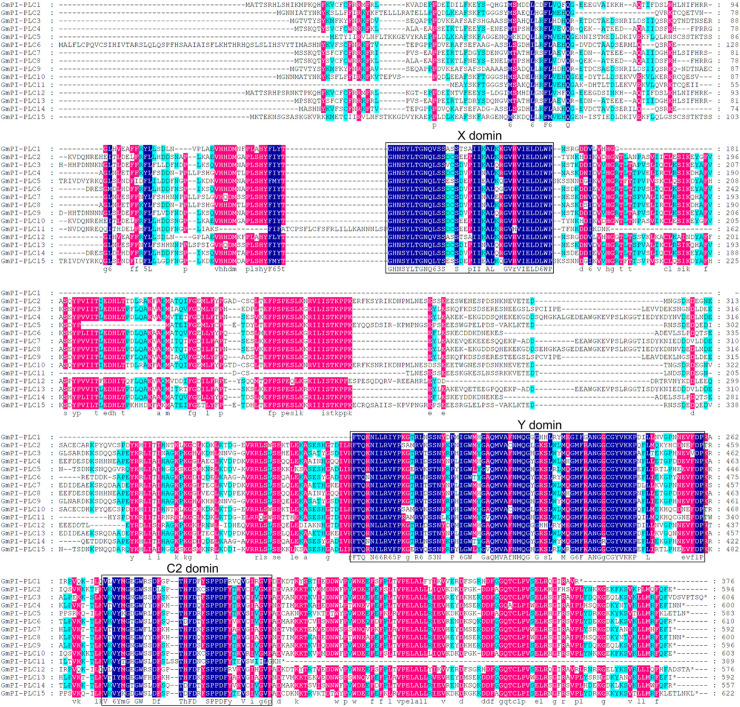
Multiple sequence alignment of 15 GmPI-PLC proteins from soybean using DNAMAN. Navy blue, red and light blue shading, respectively, represent amino acids with 100, >75, and 50% similarity of amino acids.

**FIGURE 4:**
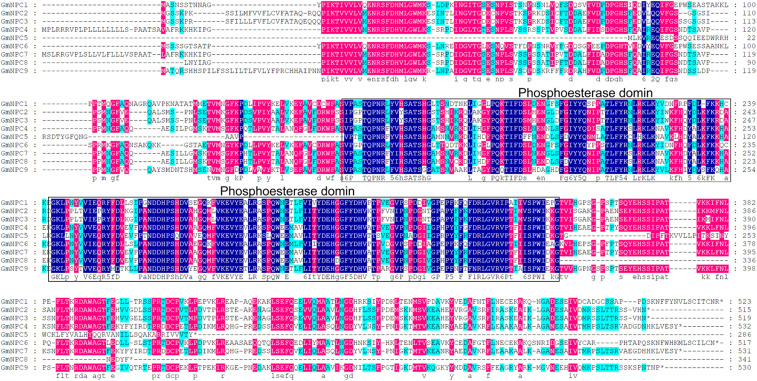
Multiple sequence alignment of nine GmNPC proteins from soybean by DNAMAN. Navy blue, red and light-blue shading, respectively, represent amino acids with 100, >75, and 50% similarity of amino acids.

The three conserved regions of the GmPI-PLC proteins were distributed on the front, middle, and rear segments of the protein sequence, and the conserved regions of GmNPCs were distributed in the middle of the amino acid sequences (Figures 3, 4).

### Gene Structure Analysis of *GmPLCs* in Soybean

**FIGURE 5:**
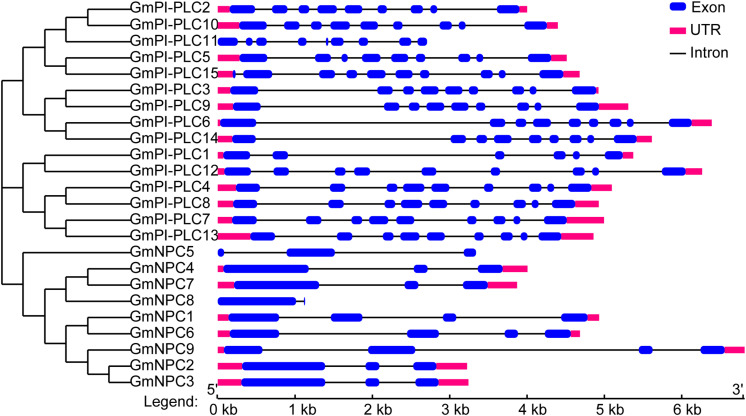
Structural analysis of *GmPLC* genes. The phylogenetic tree was constructed via MEGA7.0 software; the different classes of *GmPLC* genes make up separate clades. Introns and exons are indicated by black lines and blue boxes, respectively. The lengths of introns and exons of each gene are displayed proportionally.

**FIGURE 6:**
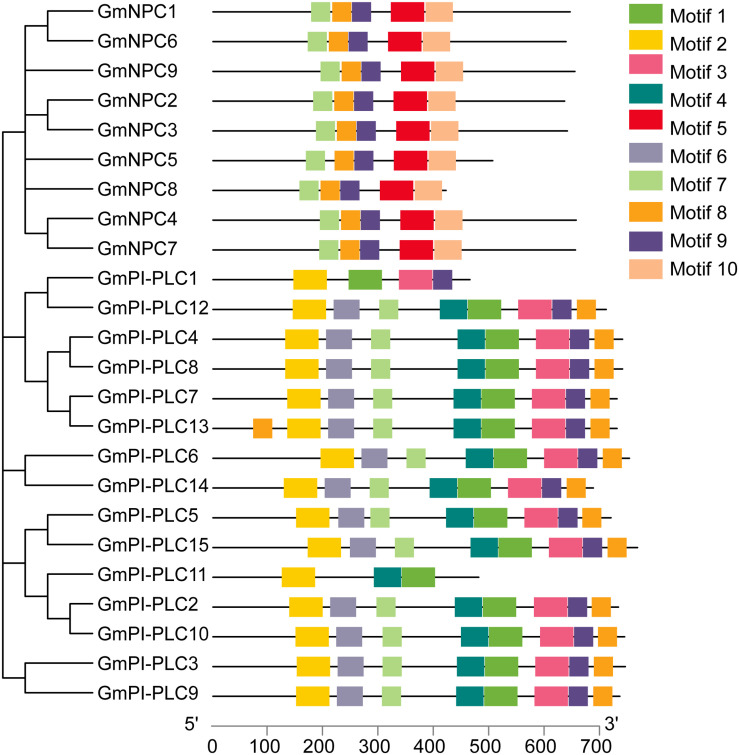
Schematic representation of the 10 conserved motifs in GmPLC proteins. Putative motifs of each GmPLC gene were determined by the MEME program and TBtools software. Each motif is indicated by a different colored box. The lengths of proteins can be estimated using the scale at the bottom.

Research suggests that the evolution of multi-gene families leads to diversity in genetic structure. The exon/intron structures of *GmPLC* genes are shown in Figure 6. The exon-intron organization of the 24 GmPLC genes was examined to obtain information on the structure, diversity, and evolution of the PLC families in soybean. The PLC family members of soybean all contained nine exons except for *GmPLC1*, and the number of introns in NPC family members were from 2–4 (Figure 5 and Supplementary Table 2).

### Analysis of GmPLC Motif Sequences in Soybean

In addition to the exon/intron pattern, conserved motifs could also be important for the various functions of *GmPLC* genes. We determined 10 different conserved motifs (Figure 6), and the results demonstrated that genes members of the same subfamily shared an analogous motif structure. The amounts of each conserved motif are shown in Supplementary Table 2. Notably, the *GmPI-PLC* genes had more motifs than the *GmNPC* genes had, and the same regions often were composed of proteins with higher homology, which corresponds to the phylogenetic analysis.

### Tissue-Specific Expression Patterns of *GmPLC* Genes in Soybean

**FIGURE 7:**
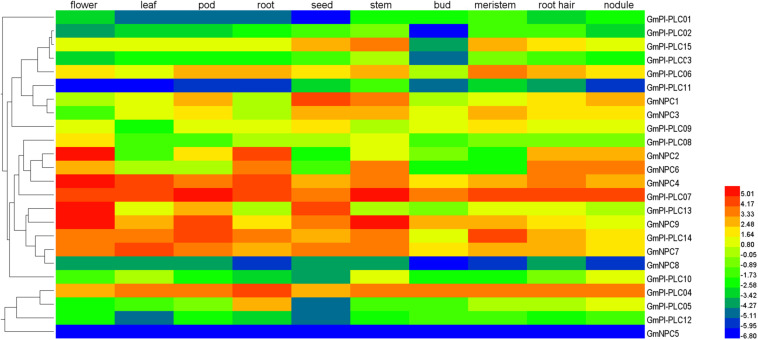
Tissue-specific expression patterns of *GmPLC* genes in different soybean tissues: flower, leaves, pod, root, seed, stem, bud, meristem, root hairs, and nodules. Color bar at the top represents log2 (FPKM) expression values where low to high expression values are, respectively, represented by blue to red colors.

To gain insight into the gene expression patterns in soybean growth and development, we used the relative transcript abundance of the *GmPLC* genes in 10 types of tissues (flower, leaf, pod, root, seed, stem, bud, meristem, root hair, and nodule) from publicly available transcriptome data in the Phytozome database. *GmPLCs* transcripts were detected in the various tissues (Figure 7 and Supplementary Table 4). A total of 24 *GmPLC* genes were expressed in tissues, whereas *GmPI-PLC7* were expressed in all selected tissues. Additionally, the expression patterns among the *GmPLCs* differed in the same tissues. For example, *GmPI-PLC4*, *GmPI-PLC7*, *GmPI-PLC14*, *GmNPC4*, and *GmNPC7* were highly expressed in most tissues. The expression of *GmPI-PLC13*, *GmNPC2* and *GmNPC9* were high in flowers, *GmNPC2* was expressed strongly in roots, and *GmPI-PLC9* and *GmNPC9* transcription were enriched in pods. The expression of *GmPI-PLC1*, *GmPI-PLC11*, *GmNPC5*, and *GmNPC8* were low in all tissues. These transcriptional patterns demonstrated that the expression of these genes might be governed by diverse and potentially tissue-dependent regulatory mechanisms.

### Expression Pattern Analysis of GmPLC Genes and Abiotic Stress

**FIGURE 8:**
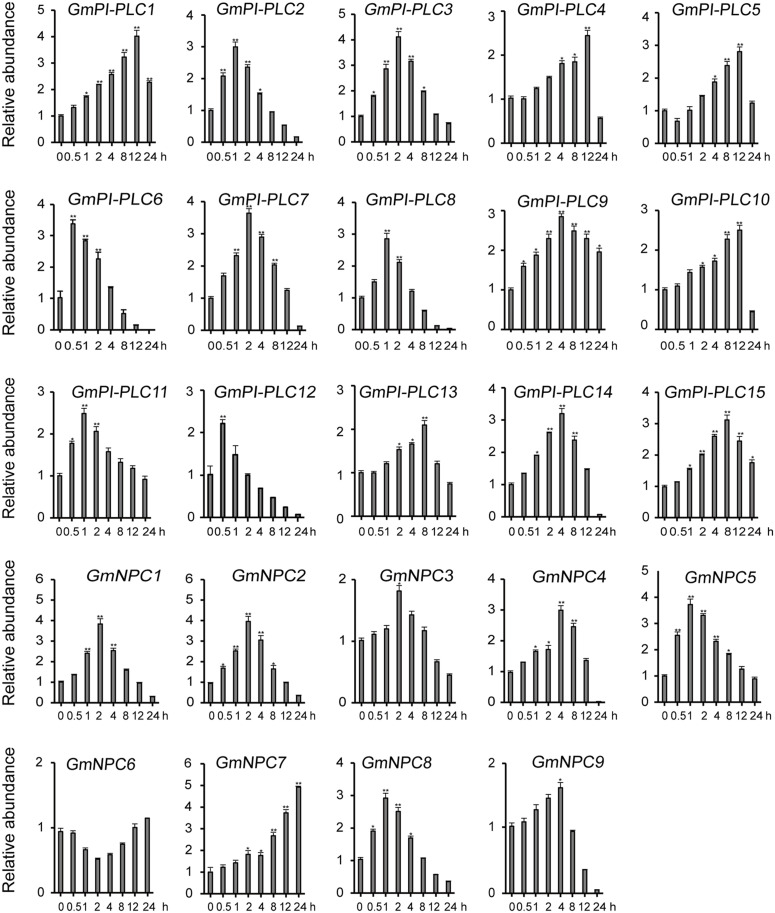
Expression patterns of all *GmPLC* genes in response to drought treatment obtained by qRT-PCR. The actin gene was used as an internal control. The data shown are means ± SD obtained from three biological replicates. ANOVA was used to determine significant differences (*P < 0.05, **P < 0.01).

**FIGURE 9:**
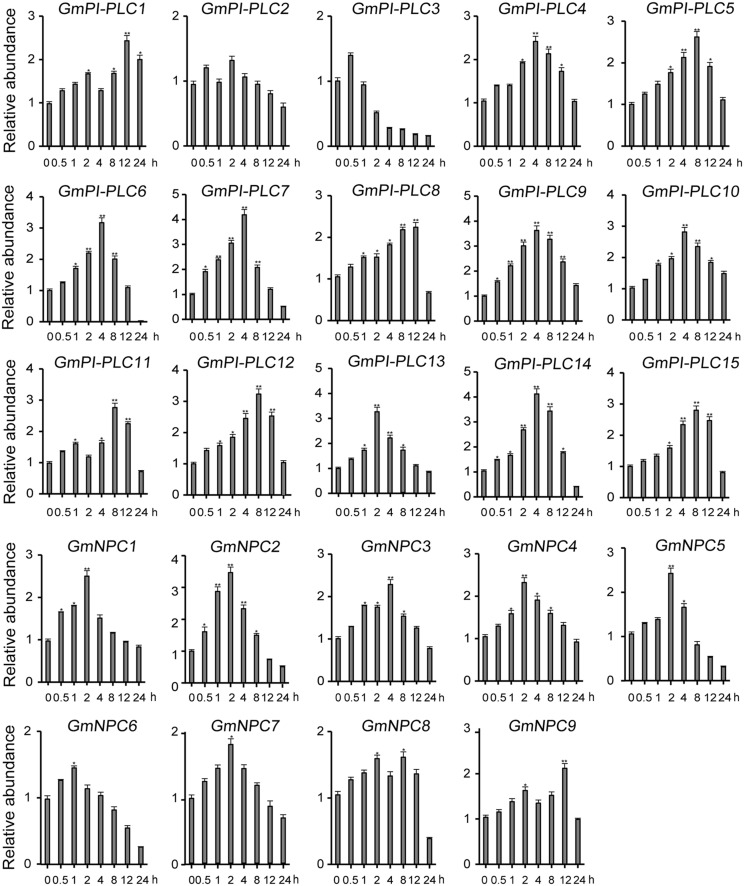
Expression patterns of all *GmPLC* genes in response to mannitol treatment obtained by qRT-PCR. The actin gene was used as an internal control. The data are means ± SD obtained from three biological replicates. ANOVA was used to determine significant differences (*P < 0.05, **P < 0.01).

**FIGURE 10:**
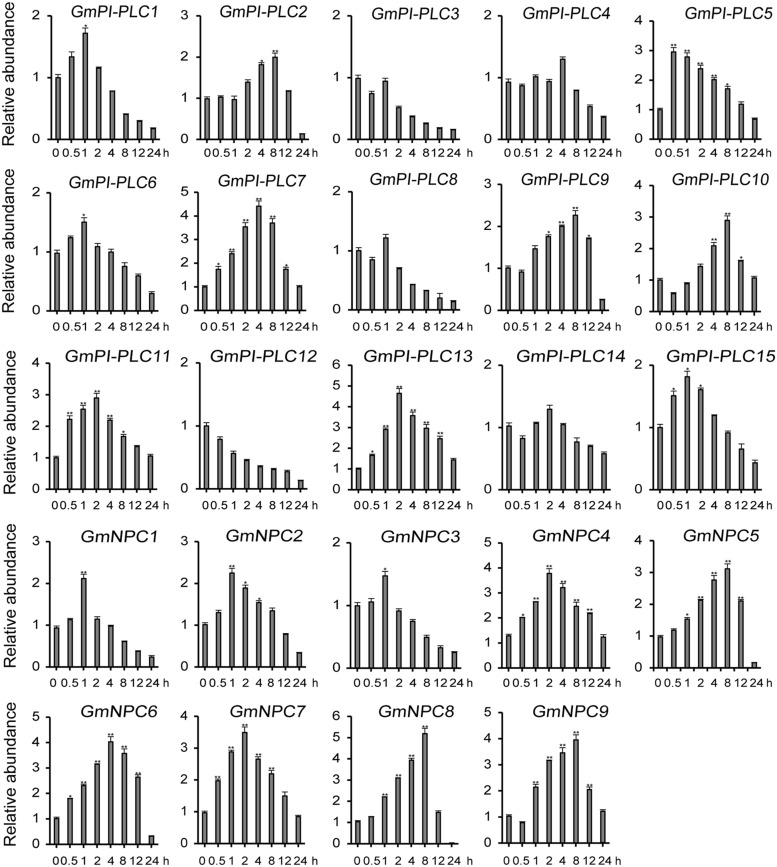
Expression patterns of all *GmPLC* genes in response to melatonin treatment obtained by qRT-PCR. The actin gene was used as an internal control. The data are means ± SD obtained from three biological replicates. ANOVA was used to determine significant differences (*P < 0.05, **P < 0.01).

The analysis of qRT-PCR results show that the expression patterns of GmPLC genes varied in response to an abiotic stress of drought, mannitol or melatonin treatment. Among the varied responses of genes in the *GmPLC* gene families, *GmPI-PLC6*, *GmPI-PLC7*, *GmNPC1*, *GmNPC2*, *GmNPC4*, *GmNPC6*, and *GmNPC7* expression increased at the early stage and then declined at the later stage of treatment (Treatment response was divided into stages, “early” was 1–2 h, “mid” was 4–8 h, and “late” was 9–12 h after initiation treatment.). Under drought stress, the transcription of *GmPI-PLC6*, *GmPI-PLC7*, *GmNPC1*, *GmNPC2*, *GmNPC5*, and *GmNPC7* were up-regulated by 3–5-fold (Figure 8). Mannitol treatment significantly induced the transcription of four GmPLC genes at 4 h after treatment; the expression of *GmPI-PLC6*, *GmPI-PLC7*, *GmPI-PLC9* and *GmPI-PLC14* were upregulated by 3–5-fold (Figure 9). With regard to the melatonin treatment, three GmPLCs (*GmPI-PLC13*, *GmNPC4*, and *GmNPC7*), two GmPLCs (*GmPI-PLC7* and *GmNPC6*) and five GmPLCs (*GmPI-PLC10*, *GmNPC5*, *GmNPC6*, *GmNPC8*, and *GmNPC9*) were most strongly upregulated (>three-fold change) at 2, 4, and 8 h, respectively (Figure 10). The expression level of *GmPI-PLC7* increased under the drought, mannitol and melatonin treatments (3. 77-, 4. 21-, and 4.52-fold, respectively).

### *GmPI-PLC7* Improved Drought Tolerance in Soybean Transformants

**FIGURE 11:**
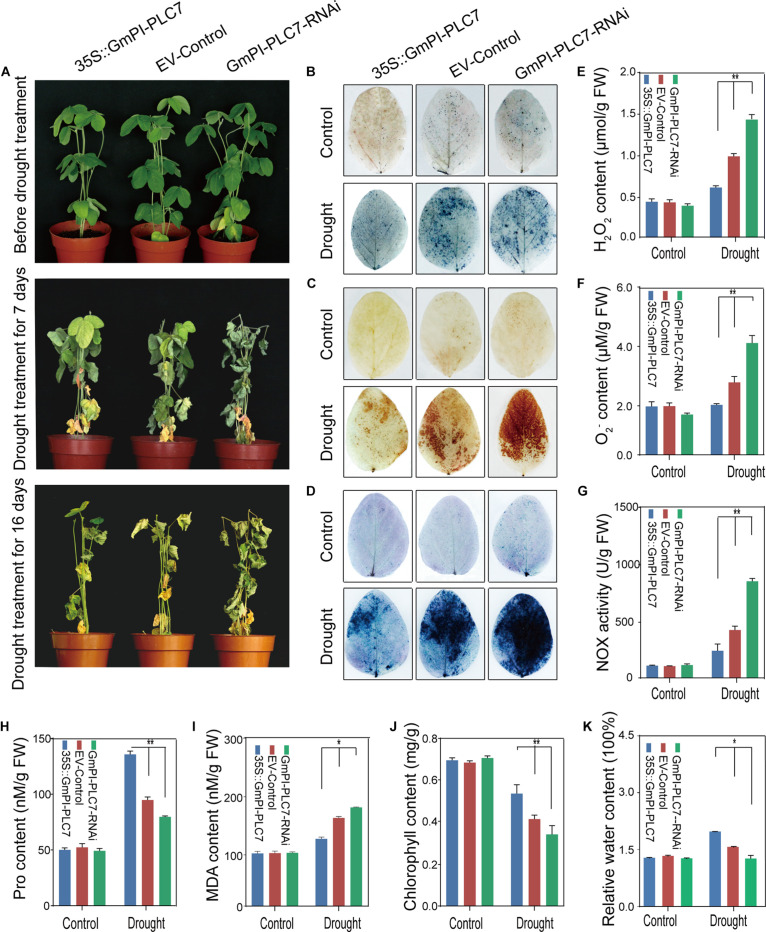
*GmPI-PLC7* improves drought tolerance in transgenic soybean hairy roots. Images of salinity-resistant phenotypes of OE, EV and RNAi lines sampled over time while subjected to drought **(A)**. NBT **(B)** and DAB **(C)** staining of the leaves of OE, EV and RNAi lines after drought or no-drought treatment for 7 days. The depth of color shows the concentrations of H_2_O_2_ and O_2_^–^ in the leaves **(B,C)**. The contents of H_2_O_2_ **(E)** and O_2_^–^ **(F)** in the leaves of OE, EV and RNAi lines after drought or no-drought treatment for 16 days. Trypan blue staining of soybean plant leaves deprived of irrigation for a week **(D)**; dead cells can be stained, while living cells cannot. The NOX **(G)**, proline **(H)**, MDA **(I)**, chlorophyll **(J)**, and relative water contents **(K)** detected in leaves of OE, EV, and RNAi lines subjected to a 250-mM NaCl treatment or the control condition for 7 days. The data are means ± SDs of three replicates. ANOVA was used to determine significant differences (*P < 0.05, **P < 0.01).

To understand how the *GmPI-PLC7* gene affects the physiological changes of soybean under drought stress, the OE, EV and RNAi lines of soybean transformed by *A. rhizogenes* were examined. No significant differences were observed between OE, EV and RNAi lines under normal growth conditions. After drought treatment for 7 days, leaves of the RNAi lines were wilting; after 16 days of drought treatment, leaves of the EV and RNAi lines were severely dehydrated-looking (Figure 11A). The survival rates of the OE, EV and RNAi lines were 100.00, 77.33, and 33.30%, respectively (Supplementary Figure 2A). After 16 days of drought exposure, the stressed phenotypes (seedlings with wrinkled leaves) from each line were selected for DAB, NBT, and trypan blue staining, and the results showed that large amounts of reactive oxygen species (ROS) accumulated in EV and RNAi lines during drought stress compared with that of OE lines (Figures 11B–D). Additionally, H_2_O_2_, O_2_^–^ and MDA contents were significantly higher in EV and RNAi lines under drought treatment than the respective contents in OE lines (Figures 11E,F,I). Higher levels of NOX activity and relative water contents were observed in RNAi lines (Figures 11G,K) than those in OE lines. Furthermore, under drought treatment, Pro, and chlorophyll contents were lower in EV and RNAi lines than those in OE lines (Figures 11H,J). These results indicated that *GmPI-PLC7* decreased the damage caused by drought treatment and improved drought tolerance in transformed soybean seedlings.

### *GmPI-PLC7* Improved Salt Tolerance in Soybean Transformants

**FIGURE 12:**
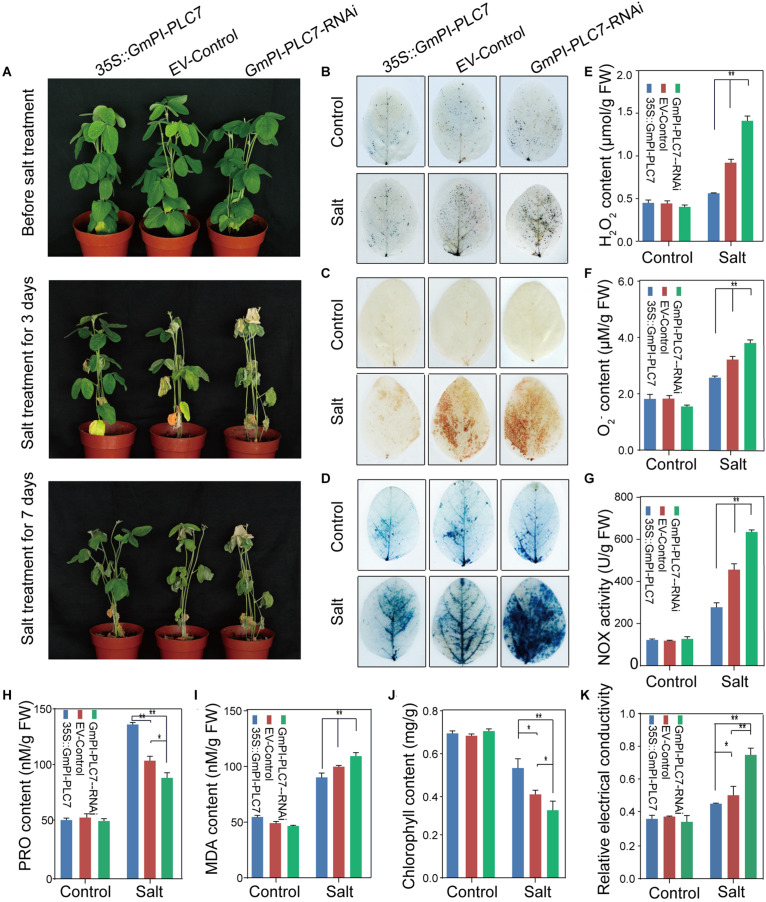
*GmPI-PLC7* improves salt-stress tolerance in transgenic soybean hairy roots. Images of salinity-resistant phenotypes of OE, EV and RNAi lines exposed to a salt treatment over time **(A)**. DAB **(B)** and NBT **(C)** staining of leaves of OE, EV and RNAi lines after the 250-mM NaCl or control treatment for 3 days. The depth of color shows the concentrations of H_2_O_2_ and O_2_^–^ in the leaves **(B,C)**. The contents of H_2_O_2_ **(E)** and O_2_^–^ **(F)** in the leaves of OE, EV and RNAi lines after the 250-mM NaCl or control treatment for 3 days. Trypan blue staining of soybean plant leaves deprived of irrigation for a week **(D)**; dead cells can be stained, while living cells cannot. The NOX **(G)**, proline **(H)** and MDA **(I)**, chlorophyll **(J)**, and relative electrical conductivity **(K)** detected in leaves of OE, EV and RNAi lines exposed to the 250-mM NaCl or control treatment 3 days. The data are means ± SDs of three replicates. ANOVA was used to determine significant differences (*P < 0.05, **P < 0.01).

The OE, EV, and RNAi lines were used to further determine the role of *GmPI-PLC7* in stress response to salt treatment. No significant differences were observed between OE, EV and RNAi lines under normal growth conditions. Under the 250-mM NaCl treatment, the RNAi lines showed stressed phenotypes of wrinkled leaves after 7 days of salt treatment, and severely dehydrated leaves were observed in both EV and RNAi lines after 7 days of salt treatment (Figure 12A). The survival rates of the OE, EV, and RNAi lines were 100.00, 67.33, and 40%, respectively (Supplementary Figure 2C). After 7 days of stress treatment, each line was DAB-, NBT-, and trypan blue-stained to evaluate the accumulation of ROS. The results showed that greater amounts of ROS accumulated in EV and RNAi lines than compared with amounts accumulated in OE lines during the salt stress (Figures 12B–D). Additionally, the H_2_O_2_, O_2_^–^ and MDA contents were significantly higher in EV and RNAi lines than in OE lines under salt stress (Figures 12E,F,I). Meanwhile, levels of NOX activity were higher in EV and RNAi lines (Figure 12G) than in OE lines. Moreover, the Pro, chlorophyll, and relative electrical conductivity levels were significantly lower in EV and RNAi lines than in OE lines (Figures 12H,J,K). Collectively, these results indicated that OE of *GmPI-PLC7* lead to greater salt tolerance, whereas RNAi lines resulted in salt sensitivity, suggesting that *GmPI-PLC7* plays a positive regulatory role in salt-stress response in soybean seedlings (Supplementary Figure 2B,D).

### *GmPI-PLC7* Activated Stress-Responsive Genes in Soybean

**FIGURE 13:**
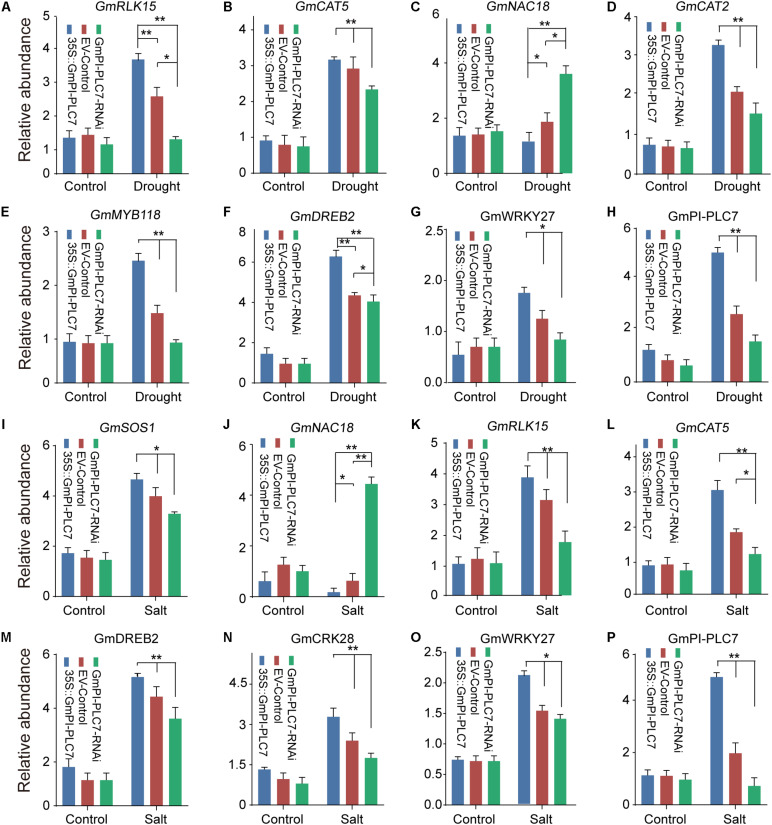
*GmPI-PLC7* regulates stress-responsive gene expression in transgenic soybean plants. The expression levels of stress-related genes in the transgenic soybean plants under drought stress **(A–G)**, respectively. The expression levels of stress-related genes in the transgenic soybean plants under salt stress **(I–O)**, respectively. The expression levels of *GmPI-PLC7* in the transgenic soybean plants under drought stress **(H)**. The expression levels of *GmPI-PLC7* in the transgenic soybean plants under salt stress **(P)**. The data are means ± SDs of three replicates. ANOVA was used to determine significant differences (*P < 0.05, **P < 0.01).

To elucidate the possible molecular mechanisms of *GmPI-PLC7* in stress responses, the expression of drought- and salt-responsive marker genes *GmMAPK15*, *GmZIP2*, *GmRLK15*, *GmCAT5*, *GmNHX1*, *GmNAC18*, *GmCAT2*, *GmRD29A*, *GmSOS1*, *GmMYB118*, *GmDREB2*, and *GmCRK28* were investigated in GmPI-PLC7-OE, EV-control and GmPI-PLC7-RNAi lines. Analysis of qRT-PCR data revealed that there were no significant differences in expression levels of all stress-responsive genes between the OE, EV and RNAi lines under normal growth conditions (Figures 13A–P). Under drought conditions, the expression levels of *GmRLK15*, *GmCAT5*, *GmCAT2*, *GmMYB118*, *GmDREB2*, and *GmWRKY27* in OE lines were significantly higher than those in EV and RNAi lines (Figures 13A–G). And the transcriptional expression level of *GmNAC18* was significantly greater in EV and RNAi lines than in OE lines. In addition, over-expressed *GmPI-PLC7* can increase the expression levels of *GmSOS1*, *GmRLK15*, *GmCAT5*, *GmDREB2*, *GmCRK28*, and *WRKY27* in seedlings exposed to salt stress, but the expression of *GmNAC18* was significantly suppressed in the OE lines and markedly increased in the RNAi lines compared to that of the EV lines (Figures 1I–O). These results indicated that overexpression of *GmPI-PLC7* may activate the expression of drought- or salt-responsive genes to meditate stress responses; however, the underlying molecular mechanism needs to be further explored (Supplementary Figure 3).

## Discussion

The PLC family of phospholipid hydrolases were first discovered in animals (Mayer et al., 1988). In recent years, PLCs have been extensively studied in various plants. This paper aimed to identify *GmPLC* members and revealed 24 PLC genes in soybean. *GmPLC* genes can be divided into two groups, PI-PLC and NPC, and PI-PLCs are reported as the major members of PLCs in plants (Kopka, 1998; Tasma et al., 2008; Nakamura and Sano, 2009). Our multiple-sequence analysis revealed that the characteristic PI-PLC-X and PI-PLC-Y domains and phospholipid-binding C2 domain were present in all *GmPI-PLC* members except *GmPI-PLC11*. Notably, an EF hand-like motif was found in five *GmPI-PLC* members (*GmPI-PLC1*, *GmPI-PLC4*, *GmPI-PLC7*, *GmPI-PLC8*, and *GmPI-PLC13*). Studies have shown that EF-hand motifs and the C2 domain may interact to facilitate membrane targeting and catalytic activity (Nomikos et al., 2015).

Intron gains and losses occur at low rates during the evolution of genomesand have significant impacts on structural and functional differentiation (Xu and Dunbrack, 2012). A previous study revealed that intron losses exceeded intron gains in rice and *Arabidopsis* at low rates (Roy and Penny, 2007). Structural analysis of *GmPLCs* revealed that all but one (*GmPI-PLC1*) of the PI-PLC members contained nine introns, and the intron-exon pattern was conserved in soybean. NPC members contained 2–4 introns in both soybean and *Arabidopsis*. Members of NPCs have been divided into two groups (I and II) based on the number of introns in *Arabidopsis* (Xu and Dunbrack, 2012); however, the two groups were not observed in GmNPCs. In brief, GmPLCs in the same clade have similar intron-exon combinations, which were similar to the findings of *Arabidopsis* and rice studies, suggesting that PLCs may perform similar functions across species. A limitation of this study is that only 24 genes were used in our analysis so the conclusions cannot fully elucidate the evolutionary characteristics of the introns. Therefore, there is still much room for improvement in determining the mechanisms of intron evolution in PLC.

The expression of genes usually reflects the potential functions of the genes. For example, the expression of the *AtPI-PLC2* gene is involved in reproductive organ development (Li et al., 2015), and the expression of the *AtPI-PLC3*, *AtPI-PLC9*, and *AtNPC1* genes are important for heat tolerance (Kang et al., 2014; Pejchar et al., 2015), whereas the *AtNPC4* and *AtNPC5* genes are essential for salt stress response (Kocourková et al., 2011; Peters et al., 2014). In rice, *OsPI-PLC1* is associated with disease resistance and *SIPLC4/6* have distinct functions in Cf-4-mediated disease resistance (Song and Goodman, 2002). These studies indicate that the expression of the large number of PLCs could be induced by hormones, chemicals, environmental stresses and pathogen infection.

The expression patterns of GmPLCs in different tissues in response to abiotic stress conditions have been studied in several species. Previous studies reported that different *PLC* genes were differentially expressed and played diverse physiological roles. For example, the expression levels of *StPLC1* and *StPLC2* in potato leaves increased significantly, while the level of *StPLC3* remained unaltered (Kopka, 1998). GmPI-PLCs were clearly expressed in various tissues and significantly increased under various stresses (Figure 7), indicating that *PI-PLCs* may play important roles in plant growth and abiotic stress-response. Our results matched those observed in earlier studies where most *GmPLCs* exhibited only background levels of expression under normal abiotic conditions, while the same *GmPLCs* could be induced by a variety of abiotic stresses. For example, *GmPI-PLC1*, *GmPI-PLC3*, and *GmNPC8* were only slightly expressed in normal conditions, while high expression levels were observed after drought treatment. In addition, the expression level of *GmPI-PLC7* increased significantly due to the drought, mannitol and melatonin treatments (Figures 8–10).

Gene duplication is a major mechanism in expanding functional diversity, which facilitates plant adaptations to their diverse environments (Schmutz et al., 2010), and functionally different duplicated-genes are more likely to be preserved during evolution (Schlueter et al., 2007). In addition, tandemly duplicated genes can produce rapid differentiation in gene expression, while some duplicated genes tend to maintain the same expression patterns (Ganko et al., 2007). Our results showed that duplicated genes exhibited diverse expression patterns (Figures 8–10).

Previous studies showed that PLC’s involvement in plant development and ABA signaling, in Arabidopsis, *AtPLC3* improved the drought tolerance by promoting root development, seed germination and stomatal opening (Zhang et al., 2018). In addition, ABA signaling occurring at the plasma membrane may be mediated by C2 domain proteins (Rodriguez et al., 2014). In this study, the C2 domain was identified in *GmPI-PLCs*, which showed that *GmPI-PLCs* may have participated in the regulation of the ABA signaling pathway in soybean. However, osmotic and temperature stresses can also induce various lipid signals (Hou et al., 2016). In the salt overly sensitive (SOS) pathway, the cytosolic Ca^2+^ signal was detected by a calcium-binding protein named EF-hand SOS3 in plants exposed to salt treatment (Xiong et al., 2002). In our study, the *GmPI-PLC7* genes have an EF-hand domain, therefore we inferred that the PLC genes may improve soybean tolerance to salt stress by participating in the SOS signaling pathway (Xiong et al., 2002). In *Arabidopsis*, background expression levels of *DREB2* genes were negatively regulated by *PI-PLC* (Ruelland et al., 2013). In our study, the analysis of *cis*-acting elements suggested that the PLC genes might be involved in responses to abiotic stresses (Supplementary Figure 4). The water loss measurements of detached leaves as ABA-related experimental data proved that GmPI-PLC7 improves the drought tolerance of soybeans (Figure 11K). It is worth noting that the expression of *GmDREB2* were induced by both ABA-independent and ABA-dependent regulatory pathways (Chen et al., 2007). *GmNAC* was induced by dehydration and ABA, and regulated drought response in an ABA-dependent manner (Tran et al., 2009). The transcript levels of 13 known abiotic stress-responsive genes (Related to drought, salt, and ABA) were analyzed in our transgenic and control soybean plants under the drought- and salt-stress treatment (Figure 13). Among them, transcription of *GmRLK15*, *GmCAT5*, *GmNAC18*, *GmCAT2*, *GmDREB2*, and *GmWRKY27* were significantly higher than that in the control lines under drought stress, while transcription of *GmRLK15*, *GmCAT5*, *GmNAC18*, *GmSOS1*, *GmDREB2*, *GmCRK28*, and *WRKY27* were clearly higher than that in the control lines under salt stress. These results altogether showed that *GmPI-PLC7* participated in soybean response to abiotic stresses.

In summary, *GmPI-PLC7* may enhance drought and salt tolerance in soybean through the ABA signaling pathway and the SOS-related calcium-signaling pathway. Fully elucidating its role in these pathways still requires further research.

## Conclusion

In our study, 15 *GmPI-PLC* genes and 9 *GmNPC* genes were identified in soybean. *GmPI-PLC7* enhanced the drought and salt tolerance of soybeans most likely through the ABA signaling pathway and the SOS-related Ca^2+^-signaling pathway, respectively.

## Data Availability Statement

The original contributions presented in the study are included in the article/Supplementary Material, further inquiries can be directed to the corresponding author/s.

## Author Contributions

Z-SX coordinated the project, and conceived and designed experiments. Z-FC performed experiments and wrote the first draft. YD and J-NR conducted the bioinformatic work and performed experiments. JC, Y-BZ, and MC provided analytical tools and managed reagents. G-ZS, X-HZ, and Y-ZM contributed with valuable discussions. All authors have read and approved the final manuscript.

## Conflict of Interest

The authors declare that the research was conducted in the absence of any commercial or financial relationships that could be construed as a potential conflict of interest.

## Supplementary Material

Supplementary Figure 1 Phylogenetic analyses of PLC proteins from *Arabidopsis*, rice, and soybean. Each clade is marked by a separate color.

Supplementary Figure 2 Measures of stress-related physiological indicators.

Supplementary Figure 3 *GmPI-PLC7* regulates stress-responsive gene expression in transgenic soybean plants.

Supplementary Figure 4 Putative *cis*-element in a 2-kb 5′ flanking region upstream from the start codon. Different *cis*-elements are indicated by colored symbols.

Supplementary Table 1 Phospholipase C genes in soybean. Detailed genomic information include Phytozome locus, chromosome, protein length, genomic locus (chromosomal location), ±stand, isoelectric point, molecular weight (kDa), and number of introns within ORFs of the PLC proteins.

Supplementary Table 2 Primers and sequences used in this study.

Supplementary Table 3 The IDs of PLC genes from Arabidopsis and rice.

Supplementary Table 4 The Expressions of PLC genes from soybean.

Supplementary Table 5 Downstream genes primers and sequences used in this study.

## Abbreviations

PLC: phospholipase C
PI-PLC: phosphatidylinositol-specific phospholipase C
NPC: phosphatidylcholine-hydrolyzing phospholipase C
DAB 3: 3-diaminobenzidine
NBT: nitro blue tetrazolium
qRT-PCR: quantitative Real-time polymerase chain reaction.
