# Supplementary figures and images for: Genomic-Wide Analysis of the PLC Family and Detection of GmPI-PLC7 Responses to Drought and Salt Stresses in Soybean
Source: Front Plant Sci. 2021 Mar 3;12:631470. doi: 10.3389/fpls.2021.631470 (PMC7982816; doi:10.3389/fpls.2021.631470)

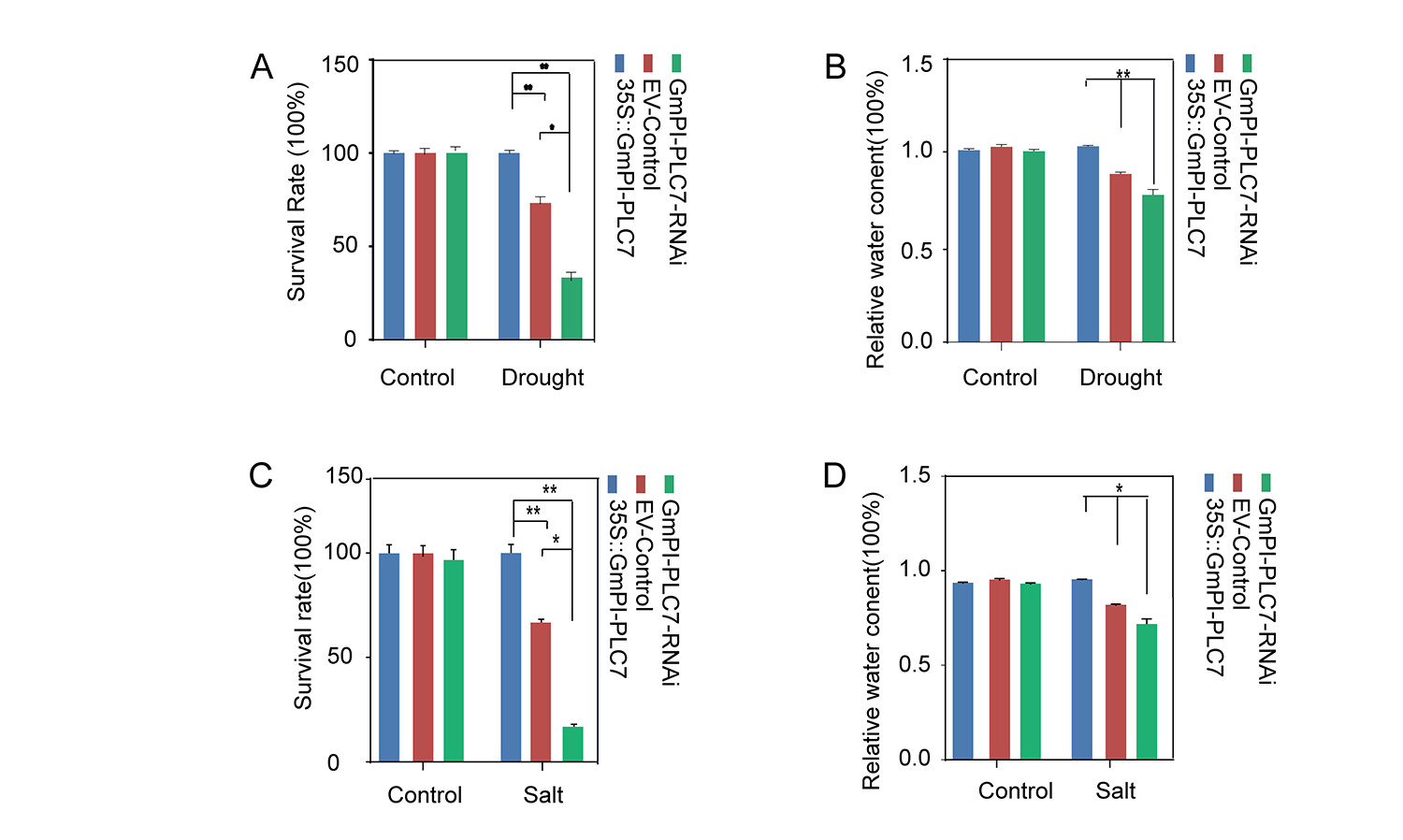

Supplement: Supplementary Figure 2 — Measures of stress-related physiological indicators. [file Image_2.TIF]

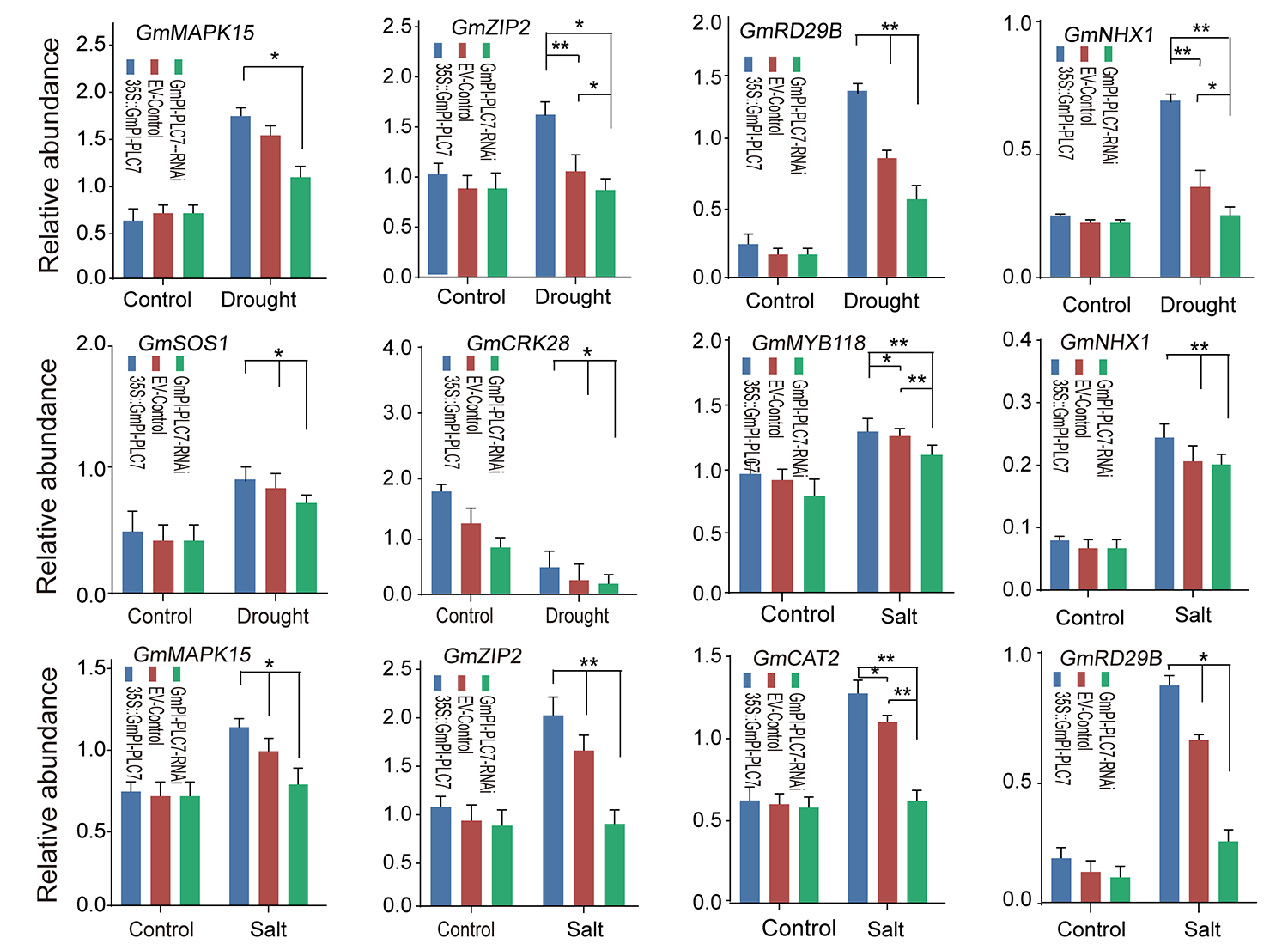

Supplement: Supplementary Figure 3 — GmPI-PLC7 regulates stress-responsive gene expression in transgenic soybean plants. [file Image_3.TIF]

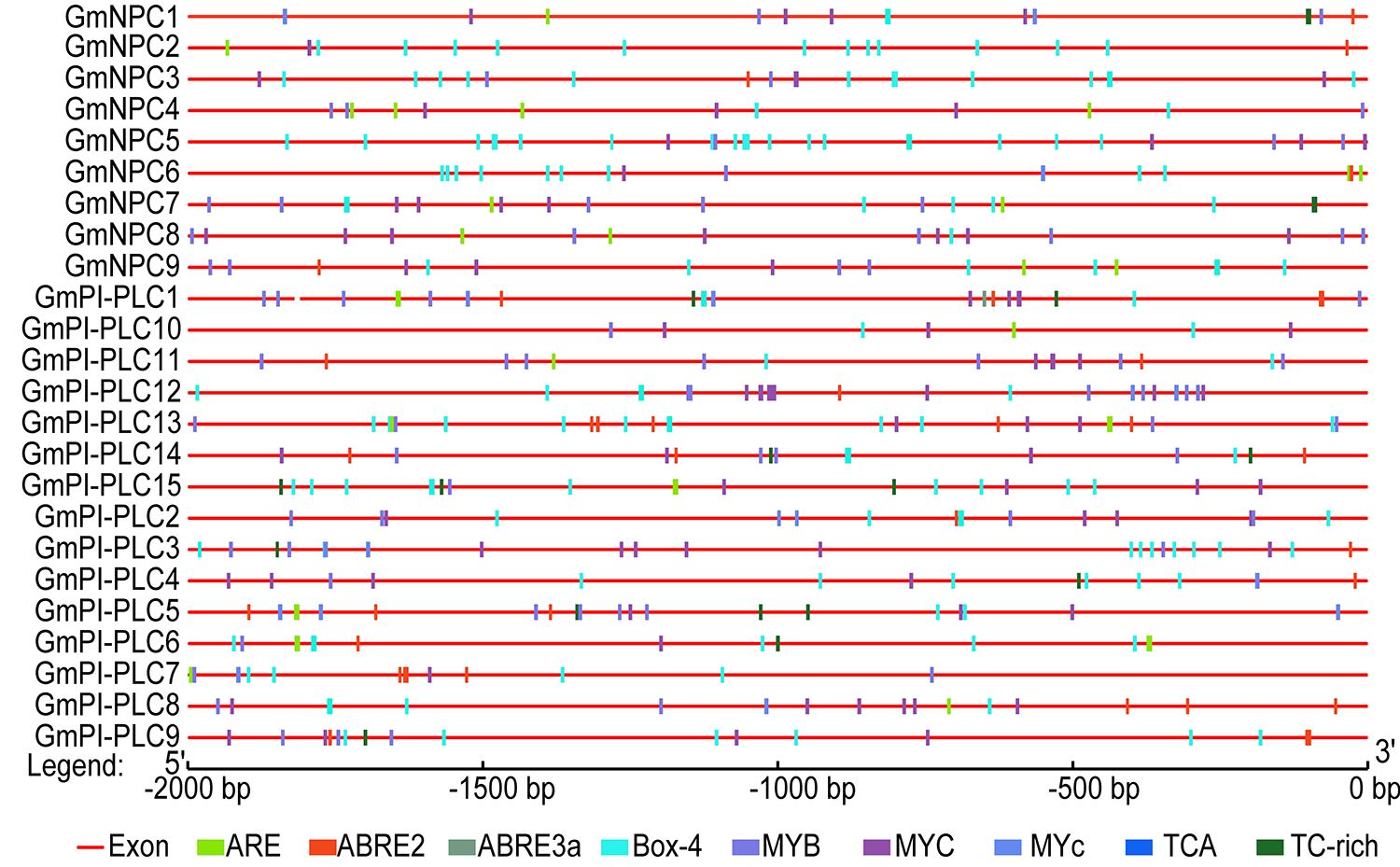

Supplement: Supplementary Figure 4 — Putative cis-element in a 2-kb 5′ flanking region upstream from the start codon. Different cis-elements are indicated by colored symbols. [file Image_4.TIF]
