# Supplementary material for: Genomic-Wide Analysis of the PLC Family and Detection of GmPI-PLC7 Responses to Drought and Salt Stresses in Soybean
Source: Front Plant Sci. 2021 Mar 3;12:631470. doi: 10.3389/fpls.2021.631470 (PMC7982816; doi:10.3389/fpls.2021.631470)
Supplement: Supplementary Table 1 — Phospholipase C genes in soybean. Detailed genomic information include Phytozome locus, chromosome, protein length, genomic locus (chromosomal location), ±stand, isoelectric point, molecular weight (kDa), and number of introns within ORFs of the PLC proteins. [file Table_1.docx]

| Table S1 \| Molecular characteristics of PLC in soybean. | | | | | | | | | |
| --- | --- | --- | --- | --- | --- | --- | --- | --- | --- |
| Name | Photozome Locus | Chromosome | Protein | Start | Stop | +/- stand | MW | Exon |  |
| GmPI-PLC1 | Glyma.02G226800 | 2 | 376 | 41369419 | 41374792 | + | 43105.31 | 6 |  |
| GmPI-PLC2 | Glyma.02G257000 | 2 | 594 | 44355260 | 44359260 | - | 68100.8 | 9 |  |
| GmPI-PLC3 | Glyma.02G257100 | 2 | 604 | 44362819 | 44367742 | - | 68719.77 | 9 |  |
| GmPI-PLC4 | Glyma.02G257200 | 2 | 600 | 44375339 | 44380435 | - | 68798.87 | 9 |  |
| GmPI-PLC5 | Glyma.11G229900 | 11 | 584 | 32521409 | 32525438 | + | 66960.54 | 9 |  |
| GmPI-PLC6 | Glyma.11G230000 | 11 | 611 | 32527636 | 32534017 | - | 69023.54 | 9 |  |
| GmPI-PLC7 | Glyma.11G230100 | 11 | 592 | 32536250 | 32541244 | - | 67293.29 | 9 |  |
| GmPI-PLC8 | Glyma.14G059200 | 14 | 600 | 4790214 | 4795140 | - | 68696.68 | 9 |  |
| GmPI-PLC9 | Glyma.14G059300 | 14 | 596 | 4799518 | 4804826 | - | 67676.72 | 9 |  |
| GmPI-PLC10 | Glyma.14G059400 | 14 | 603 | 4808851 | 4813248 | - | 69262.88 | 9 |  |
| GmPI-PLC11 | Glyma.14G059700 | 14 | 389 | 4825060 | 4827766 | + | 44628.65 | 9 |  |
| GmPI-PLC12 | Glyma.14G193800 | 14 | 629 | 45860185 | 45866449 | - | 66383.3 | 9 |  |
| GmPI-PLC13 | Glyma.18G027100 | 18 | 592 | 2033839 | 2038697 | - | 67561.63 | 9 |  |
| GmPI-PLC14 | Glyma.18G027200 | 18 | 556 | 2042578 | 2048191 | - | 63343.64 | 9 |  |
| GmPI-PLC15 | Glyma.18G027300 | 18 | 622 | 2049280 | 2053959 | + | 70939.12 | 9 |  |
| GmNPC1 | Glyma.03G092800 | 3 | 571 | 27508753 | 27513684 | + | 58369.81 | 4 |  |
| GmNPC2 | Glyma.04G196700 | 4 | 593 | 46848426 | 46851649 | - | 59503.73 | 3 |  |
| GmNPC3 | Glyma.06G169100 | 6 | 590 | 14124541 | 14127782 | - | 33387.14 | 3 |  |
| GmNPC4 | Glyma.11G178400 | 11 | 801 | 23616689 | 23620694 | - | 57505.39 | 3 |  |
| GmNPC5 | Glyma.15G188700 | 15 | 311 | 19743566 | 19746905 | - | 59559.21 | 3 |  |
| GmNPC6 | Glyma.16G081200 | 16 | 568 | 8706286 | 8710956 | - | 59371.65 | 4 |  |
| GmNPC7 | Glyma.18G064000 | 18 | 801 | 5847976 | 5851846 | + | 58273.57 | 3 |  |
| GmNPC8 | Glyma.18G064200 | 18 | 551 | 5863321 | 5864449 | + | 38366.33 | 2 |  |
| GmNPC9 | Glyma.20G040600 | 20 | 645 | 7153509 | 7160322 | - | 58043.92 | 4 |  |
