# Supplementary material for: Genomic-Wide Analysis of the PLC Family and Detection of GmPI-PLC7 Responses to Drought and Salt Stresses in Soybean
Source: Front Plant Sci. 2021 Mar 3;12:631470. doi: 10.3389/fpls.2021.631470 (PMC7982816; doi:10.3389/fpls.2021.631470)
Supplement: Supplementary Table 2 — Primers and sequences used in this study. [file Table_2.docx]

| **Table S2**: Primers and sequences used in this study. | |
| --- | --- |
| **Primer** | **Sequence** |
| *GmPI-PLC7a-F* | 5'ACGTATCCATTCTCCGACCAA 3' |
| *GmPI-PLC7a-R* | 5'ACTCCTTGTGACAGACAGCAT 3' |
| *GmPI-PLC1-F* | 5' GACCTGTGCGATTTCTTAGTTC 3' |
| *GmPI-PLC1-R* | 5' CGAAAGAAGGCTTCAACATGAA 3' |
| *GmPI-PLC2-F* | 5' AGAGAACATGAGATCACCCTTG 3' |
| *GmPI-PLC2-R* | 5' GGAGCACCCATATCATGATGTA 3' |
| *GmPI-PLC3-F* | 5' CAATTCCTACCTGACTGGGAAT 3' |
| *GmPI-PLC3-R* | 5' ATCTAGTTCAATTACCCGCACA 3' |
| *GmPI-PLC4-F* | 5' CTCCAGACTCCAATAAGAACGA 3' |
| *GmPI-PLC4-R* | 5' TCCATGAGATTCTGAAGCCTTT 3' |
| *GmPI-PLC5-F* | 5' ATTTCAGGCCGTATATAGGGTG 3' |
| *GmPI-PLC5-R* | 5' GTGTTCGGATCAGAAATTCAGG 3' |
| *GmPI-PLC6-F* | 5' GATAAGAGTGCACCTGAGTACA 3' |
| *GmPI-PLC6-R* | 5' TTCAAGTGCTTGTTCACTCAAG 3' |
| *GmPI-PLC7-F* | 5' TGGGAACTAAGAAGTGGAATCC 3' |
| *GmPI-PLC7-R* | 5' TTCAAAGTGCATGAGAAGCTTC 3' |
| *GmPI-PLC8-F* | 5' TGCATAGCCAAAAGGGAGATAA 3' |
| *GmPI-PLC8-R* | 5' TACAAGACCAACAAGTCTCCTC 3' |
| *GmPI-PLC9-F* | 5' GTTCTTCCGTTTCTTGTTCCTC 3' |
| *GmPI-PLC9-R* | 5' GTGACAACGGAGCATTCATATC 3' |
| *GmPI-PLC10-F* | 5' ACTACACGTTATTGGATTCGGA 3' |
| *GmPI-PLC10-R* | 5' GACCGTTGGAATCATCATGAAG 3' |
| *GmPI-PLC11-F* | 5' GGTTCAGATGGTTGCATTCAAT 3' |
| *GmPI-PLC11-R* | 5' GTAGGATCAAACTCATTGCCAC 3' |
| *GmPI-PLC12-F* | 5' TAAGACAAGGAATACGTGCAGT 3' |
| *GmPI-PLC12-R* | 5' ACAGAGATTTCTATGCAGTGCT 3' |
| *GmPI-PLC13-F* | 5' AATATAAGGGTGCGAGTGAGAG 3' |
| *GmPI-PLC13-R* | 5' TTCAAAATCCGTGGAAACTCAC 3' |
| *GmPI-PLC14-F* | 5' TTCATGGAAGGACTCTTACCAC 3' |
| *GmPI-PLC14-R* | 5' GCTTGAAGATCTGGAGTAAGGT 3' |
| *GmPI-PLC15-F* | 5' TAGACGGCTAAGTTTGAGTGAG 3' |
| *GmPI-PLC15-R* | 5' CACCCTATATACGGCCTGAAAT 3' |
| *GmNPC1 F* | 5' ACATTTACGAGCAAATCTTCGG 3' |
| *GmNPC1 R* | 5' AACAGTCACAAACCGCATATTC 3' |
| *GmNPC2 F* | 5' ATGAGCAAGAAGATGAGTGTGA 3' |
| *GmNPC2 R* | 5' AGATATGCAGAGATAGCCACAC 3' |
| *GmNPC3 F* | 5' FATATCAAGAGGCAGTTAGCGAA 3' |
| *GmNPC3 R* | 5' AGTAAATCCCAAAGTCTAGGCC 3' |
| *GmNPC4 F* | 5' TCGAGTCCCTCTTCCTCTG 3' |
| *GmNPC4 R* | 5' CGATTCTCCATCACTATCACCA 3' |
| *GmNPC5 F* | 5' AATGTTCGTGAAGGAGGTGTAT 3' |
| *GmNPC5 R* | 5' GCTCATCGTACGTAATCAACAC 3' |
| **Primer** | **Sequence** |
| *GmNPC6 F* | 5' ACTGTGAAGATGCATTTGGAAC 3' |
| *GmNPC6 R* | 5' GCGGTCTAGCACAATAAACAAT 3' |
| *GmNPC7 F* | 5' TGACAGTGAAAGAAGCAAACAG 3' |
| *GmNPC7 R* | 5' TGTGACAATGGCTGACTCATTA 3' |
| *GmNPC8 F* | 5' CTCTCATTCGACGTCTACTACC 3' |
| *GmNPC8 R* | 5' AAGGCATAGTCGTGGAACTTTA 3' |
| *GmNPC9 F* | 5' CTTCGGCATCTACTACCAGAAC 3' |
| *GmNPC9 R* | 5' TGGAACTTGAGCACGTACTTTA 3' |
