# Supplementary material for: Genomic-Wide Analysis of the PLC Family and Detection of GmPI-PLC7 Responses to Drought and Salt Stresses in Soybean
Source: Front Plant Sci. 2021 Mar 3;12:631470. doi: 10.3389/fpls.2021.631470 (PMC7982816; doi:10.3389/fpls.2021.631470)
Supplement: Supplementary Table 5 — Downstream genes primers and sequences used in this study. [file Table_5.docx]

| **Table S5**: Downstream genes primers and sequences used in this study. | |
| --- | --- |
| **Primer** | **Sequence** |
| GmMYB118-F | TTGCTTGAGATTATGGTCCTTG |
| GmMYB118-R | CATCACCCTTTCCTTCAACC |
| GmRD29B-F | AAGTGAGTTGGGAGGCAGTG |
| GmRD29B-R | AAGTTCACAAACAGAGGCATCA |
| GmNAC18-F | TGCAAGGAGGAGCACAAGAG |
| GmNAC18-R | CACAGAACCCAGTCGTCCAA |
| GmMAPK15-F | GTCGCCGTCACCGAGG |
| GmMAPK15-R | CAAGCCGCCGGAGTCTATT |
| GmCRK28-F | ATGTTGTTACTCTTACTCAACTA |
| GmCRK28-R | TTATCGAGGATAAAATATACTTG |
| GmDREB2-F | AGCGAAAGCAGCAGCACC |
| GmDREB2-R | GTTAAGGCGAGCGGAAGG |
| GmWRKY27-R | CATTGGATTTGGAGGTGAAGA |
| GmWRKY27-F | TCTCTGTGAGCATTTCGGTTA |
| Gmzip2-F | TGTCGGCTTTCTGACAGTTTAT |
| Gmzip2-R | GATACGATGCAGGCGAGAAA |
| GmSOS1-F | TTGTGCTGCATTTCTTCGAG |
| GmSOS1-R | CGTGCTTCTTCTCCTTCCAC |
| GmCAT5-F | GTCGCTTGGTCCTGAATA |
| GmCAT5-R | AAGTCTGTGCCTTTGTGA |
| GmCAT-2 | GCGCTAGTGCAAAGGGTTTC |
| GmCAT-2 | AAGGTTTCAGGGCTACCACG |
| GmNHX1-F | GGGTAGTGAGAACGCTGTG |
| GmNHX1-R | ATTGTGAGATGTTTGCTGGTAATC |
| GmActin-F | ACATTGTTCTTAGTGGTGGCT |
| GmActin-R | CTGTTGGAAGGTGCTGAG |
