# Supplementary material for: Genomic-Wide Analysis of the PLC Family and Detection of GmPI-PLC7 Responses to Drought and Salt Stresses in Soybean
Source: Front Plant Sci. 2021 Mar 3;12:631470. doi: 10.3389/fpls.2021.631470 (PMC7982816; doi:10.3389/fpls.2021.631470)
Supplement: Supplementary file 10 [file Data_Sheet_1.ZIP › raw date/PCR Result.pdf]

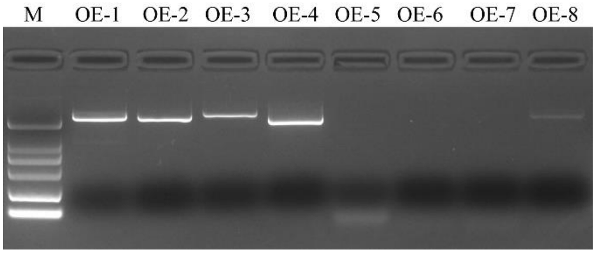

M :marker ,OE 1-4: positive lines，OE 5-8: negative strain
Figure1: GmPI-PLC7-OE 3 positive test results test results

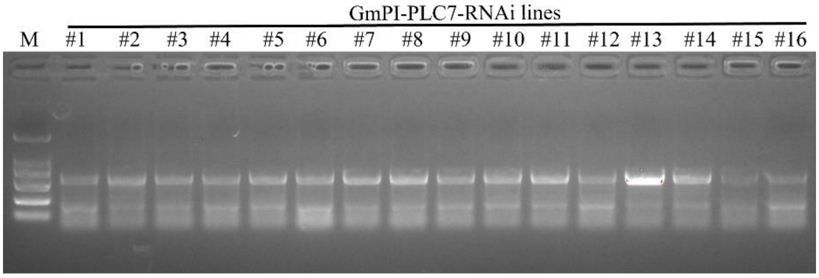

M: marker, OE 1-16: positive lines
Figure 2: GmPI-PLC7-RNAi positive test results test results
